# Therapy-induced androgen receptor signaling as a candidate upstream driver of B7-H3–linked immune exclusion in melanoma: mechanisms and translational opportunities

**DOI:** 10.3389/fmed.2026.1807470

**Published:** 2026-03-25

**Authors:** Adrian P. Mansini, John R. Hyngstrom, Kyle T. Amber

**Affiliations:** 1 Department of Dermatology, Rush University Medical Center, Chicago, IL, United States; 2 Department of Urology, Rush University Medical Center, Chicago, IL, United States; 3 Division of Surgical Oncology, Department of Surgery, Rush University Medical Center, Chicago, IL, United States

**Keywords:** androgen receptor (AR), B7-H3 (CD276), BRAF/MEK inhibitors, immune checkpoint blockade, immune exclusion, melanoma, therapy resistance

## Abstract

Melanoma frequently develops resistance to BRAF/MEK–targeted therapy and immune checkpoint blockade (ICB), often through therapy-driven tumor state transitions that include immune exclusion, transcriptional plasticity, and microenvironmental remodeling. B7-H3 (CD276) has been linked to immune-cold states and is being pursued as a therapeutic target. Yet, the upstream regulators that promote or stabilize B7-H3 immune exclusion in melanoma remain incompletely defined. Here, we propose a testable framework in which therapeutic pressure increases tumor-intrinsic androgen receptor (AR) signaling, which may promote or reinforce a B7-H3–linked immune-excluded resistance program. As a hypothesis-generating human anchor, in melanoma patients treated with anti-CTLA-4, AR and B7-H3 show no pre-treatment association, but a positive association emerges post-treatment. To avoid over-reliance on melanoma-specific preliminary observations, we integrate mechanistic precedent from other tumor contexts in which B7-H3 expression is shaped by defined signaling and epigenetic programs, including reported AR binding proximal to B7-H3 in prostate cancer and upstream control by stress- and growth-factor pathways. We then outline falsifiable mechanisms by which AR could interact with these regulatory nodes to increase B7-H3 output and barrier-like immune exclusion, and we highlight translational opportunities to therapeutically disrupt the AR–B7-H3 axis through modulation of the AR pathway and/or B7-H3–directed agents. This Perspective defines near-term experiments and study designs to validate directionality, delineate the relevant resistant tumor states, and establish a rational basis for combination therapy.

## Introduction

Despite advances in targeted therapy and immunotherapy, melanoma remains a model of therapy-driven tumor evolution. Importantly, melanoma exhibits consistent sex disparities in outcomes, with males showing poorer melanoma-specific survival than females across stages in population-based analyses (1, 2), supporting the biological plausibility that sex-linked tumor/host programs could modulate therapy response and motivating AR-centered hypotheses. BRAF/MEK inhibitors (BRAFi/MEKi) can produce rapid tumor regression in BRAF V600-mutant disease. Yet, over 80% of patients ultimately progress (3–5). Immune checkpoint blockade (ICB) yields durable responses in a subset of patients but leaves at least 40% of patients with primary or acquired resistance (4, 6, 7). These patterns suggest that melanoma resistance is frequently governed not only by isolated genetic alterations but also by coordinated tumor-state transitions that reshape both tumor-intrinsic programs and microenvironmental interactions.

A recurring feature of resistant disease is immune exclusion, characterized by limited effective cytotoxic T-cell engagement within tumor nests, together with stromal and myeloid programs that restrict immune trafficking and effector function. A clinically useful resistance framework can be assessed by identifying (i) upstream drivers that are inducible by therapy, (ii) tractable nodes linking the tumor state to immune exclusion, and (iii) practical readouts that enable patient stratification and longitudinal assessment.

B7-H3 (CD276) has attracted increasing attention as an immune-regulatory molecule enriched in immune-cold tumor states and as a targetable surface molecule across multiple cancer types (8, 9). In melanoma, B7-H3 has been associated with an “armored-cold” architecture characterized by low numbers of tumor-infiltrating lymphocytes and collagen-rich features consistent with immune exclusion (10). To contextualize the sex-linked AR hypothesis, we performed an exploratory query of publicly available The Cancer Genome Atlas SKCM bulk RNA-seq data via UALCAN (*n* = 461 samples), in which CD276 transcript levels did not differ significantly between male and female tumors (male, *n* = 286 vs. female, *n* = 175, *p* = 0.537), noting that this comparison is unadjusted for stage, purity, and sample composition (11, 12). This argues against a large constitutive baseline sex difference in CD276 in untreated bulk transcriptomes and suggests that any sex-linked effects may be context-dependent (e.g., therapy-induced state transitions and/or upstream regulators such as AR). Consistent with this framing, pan-cancer analyses report higher tumor purity and lower immune/stromal scores in males, a context that could favor immune-excluded architectures even without baseline CD276 sex differences (13). Nonetheless, the upstream signals that induce or stabilize a B7-H3–high immune-excluded state during therapy remain poorly defined.

B7-H3 (CD276) is a B7-family immune checkpoint glycoprotein whose immunologic function appears context-dependent, but in cancer, it is most often associated with suppression of effective antitumor immunity, tumor progression, and adverse clinical behavior (14, 15). Importantly, B7-H3 expression is not restricted to malignant cells; within the tumor microenvironment, it has also been described on myeloid cells, endothelial cells of the abnormal tumor vasculature, and cancer-associated fibroblasts, and can be induced in selected immune populations, such as dendritic cells and NK cells, in specific contexts (14, 16). The androgen receptor (AR), in turn, is a ligand-activated nuclear receptor transcription factor best known in prostate cancer but increasingly implicated in melanoma biology, where it has been linked to proliferation, invasiveness, metastasis, therapy resistance, and immune evasion (17). Beyond tumor cells, AR signaling can also shape immune-cell function, including effects on T cells, NK cells, and myeloid populations, generally in ways consistent with reduced antitumor immune activity (18, 19). Together, these observations provide a biologic rationale for considering a possible AR–B7-H3 axis in melanoma, particularly in treatment-adapted states in which tumor-intrinsic signaling and immune exclusion may converge.

**Figure 1:**
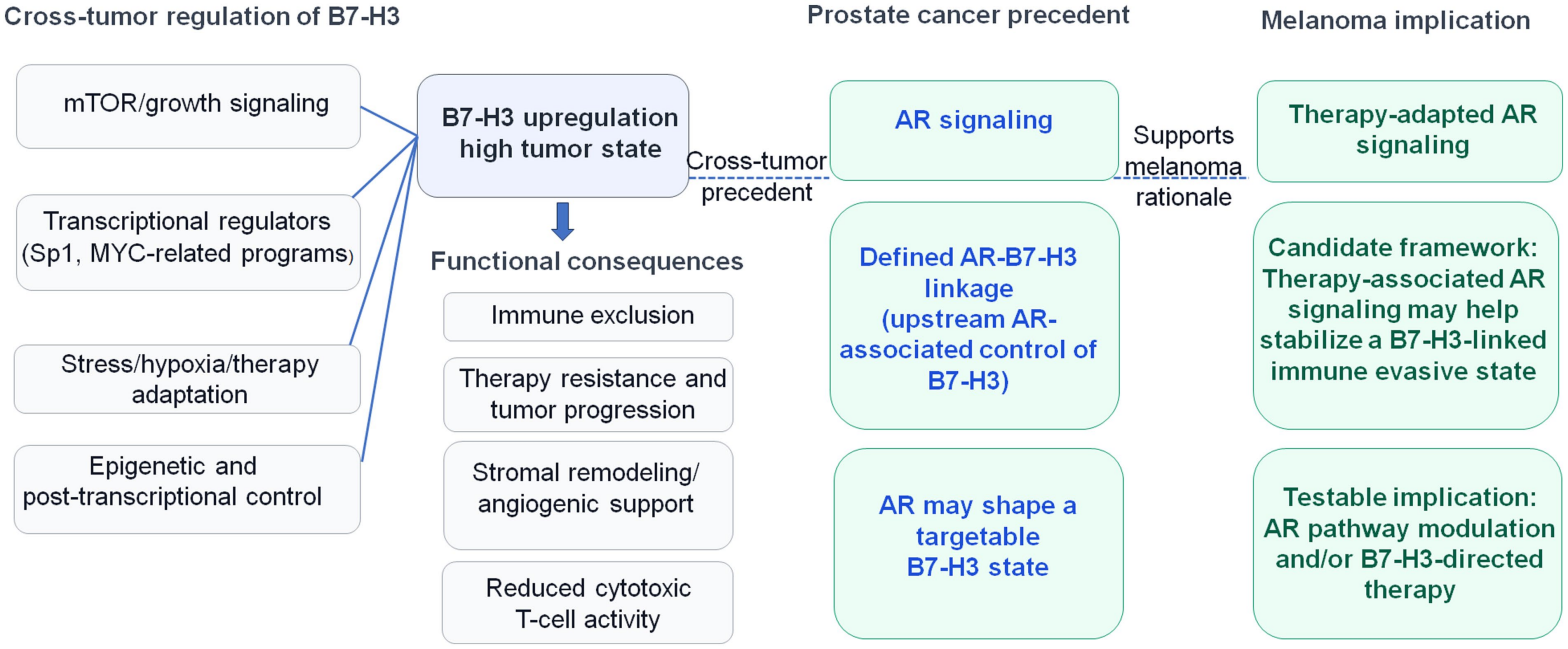
Mechanistic precedent for B7-H3 regulation across tumors and rationale for a candidate AR–B7-H3 axis in melanoma. This schematic summarizes regulatory mechanisms reported to influence B7-H3 (CD276) across tumor types, including transcriptional, signaling, and adaptive-stress pathways, and highlights a more defined AR–B7-H3 relationship in prostate cancer. Together, these observations provide mechanistic precedent for the hypothesis that therapy-associated AR signaling may contribute to a B7-H3-linked immune-evasive state in melanoma.

Building on this rationale, we hypothesize that therapy-induced AR signaling may act as an upstream regulator linking therapeutic pressure to a B7-H3–associated immune-exclusion program in melanoma. To ground this framework in established evidence from the broader field, rather than relying on melanoma-specific preliminary observations, we also summarize regulatory mechanisms of B7-H3 described in other tumor contexts and indicate where AR could plausibly intersect these pathways. We then outline the translational implications of this proposed AR → B7-H3 framework in melanoma and summarize its mechanistic basis in Figure 1.

## Therapy resistance in melanoma often converges on immune exclusion

Resistance to melanoma therapy is often framed in terms of classic pathways, such as MAPK reactivation under BRAFi/MEKi. Yet clinically resistant tumors frequently exhibit broader state transitions that encompass altered differentiation, stress adaptation, migration, and invasion, and immune interactions (20–22). These transitions can manifest as immune exclusion through convergent mechanisms, including impaired immune entry and effector function, microenvironmental remodeling that limits intratumoral immune trafficking, and engagement of alternative suppressive circuits (e.g., myeloid-dominant suppression and non–PD-1 checkpoint pathways). Therapy itself can act as a selective pressure that favors or induces these states, motivating the search for therapy-responsive upstream drivers that could be intercepted before immune exclusion becomes entrenched. These therapy-associated pressures may help explain clinically relevant differences between treatment strategies, as illustrated by the DREAMseq trial comparing upfront ICB with targeted therapy in stage IV melanoma (23).

## B7-H3 as a candidate node in immune-cold melanoma

B7-H3 is frequently expressed in tumors and has been linked to adverse clinical features across malignancies. In pan-cancer transcriptomic analyses, B7-H3–high tumors show consistent enrichment for EMT-associated programs and upstream pathways, including Wnt, Notch, and TGFβ signaling, and exhibit lower inferred CD8+ T-cell fractions—features consistent with a barrier-like architecture that may limit checkpoint blockade efficacy (24). Although its receptor biology is context-dependent and incompletely defined, multiple lines of evidence connect B7-H3 to immune evasion and tumor aggressiveness, including roles beyond canonical T-cell inhibition, such as invasion, migration, angiogenesis, and tumor-intrinsic signaling programs (8, 9). In melanoma, a B7-H3–high phenotype has been associated with immune-cold and collagen-rich “armored” states that may be difficult to reverse with standard ICB alone (10).

From a translational perspective, the attraction of B7-H3 is that it (i) tracks immune-cold architectures, (ii) is surface-accessible, and (iii) is increasingly targetable with multiple clinical-stage modalities. This framing raises a practical question: which therapy-inducible tumor-intrinsic programs contribute to B7-H3-high immune-cold architecture during adaptation? We focus on AR signaling as one plausible, testable upstream axis.

## AR signaling as a candidate upstream driver of a B7-H3–linked resistance state in melanoma

### Human data anchor: a therapy-associated AR–B7-H3 coupling signal in anti–CTLA-4–treated melanoma patients

As a clinical, hypothesis-generating anchor for this framework, we interrogated the Memorial Sloan Kettering Cancer Center (MSK) melanoma cohort of patients treated with anti–CTLA-4 therapy using cBioPortal (25, 26). To explore whether the relationship between AR and B7-H3 changes with therapy exposure, we performed separate correlation analyses in pre-treatment and post-treatment RNA-seq subsets.

In the pre-treatment RNA-seq subset, AR and B7-H3 were not positively associated at baseline (*n* = 7; Spearman *ρ* = −0.50, *p* = 0.253; Pearson *r* = −0.24, *p* = 0.606). By contrast, in the post-treatment RNA-seq subset, AR and B7-H3 showed a significant positive association (*n* = 14; Spearman *ρ* = 0.59, *p* = 0.027; Pearson *r* = 0.54, *p* = 0.045), indicating concordant results using both rank-based and linear correlation analyses.

Although limited by sample size, retrospective analysis, and incomplete RNA-seq availability, this pattern is consistent with the emergence of a therapy-associated AR–B7-H3 coupling state in melanoma. Because correlation cannot establish causality, these findings should be interpreted cautiously. Nevertheless, the observed post-treatment association provides a clinically grounded rationale for further mechanism-first studies in treatment-conditioned melanoma models and matched pre- and on-treatment patient specimens to determine when AR–B7-H3 coupling arises, whether it is directional, and how it relates to adaptive resistance and immune exclusion. Clinical characteristics of the treated patients, including sex distribution, are summarized in Table 1.

**Table 1:** Clinical characteristics of treated patients.

*N*	Sex	Age	Treatment response	M stage	Overall survival (months)	Overall survival status	Response duration (weeks)	Treatment	Tumor site
1	M	70	NR	M1c	52.8	Alive	52	Ipilimumab	Portal lymph node metastasis
2	M	59	NR	M1c	6	Deceased	13	Ipilimumab	CNS metastasis
3	M	74	ND	M1b	32.4	Deceased	12	Ipilimumab	Left arm melanoma
4	F	63	ND	M1c	8.7	Deceased	13	Ipilimumab	Small bowel metastasis
5	F	64	ND	M1c	7.2	Deceased	10	Ipilimumab	Small bowel resection
6	F	54	NR	M1c	53.9	Alive	72	Ipilimumab	Gluteal metastasis
7	F	43	NR	M1b	34.8	Deceased	68	Ipilimumab+dacarbazine	Axillary soft tissue metastasis
8	M	55	NR	M1c	14	Deceased	24	Ipilimumab	Upper back metastasis
9	M	53	NR	M1c	83.4	Alive	361	Ipilimumab	Gluteal metastasis
10	F	50	ND	M1c	24.5	Deceased	21	Ipilimumab	Skin and breast metastasis
11	F	48	R	M1c	4.8	Deceased	12	Ipilimumab	Small bowel metastasis
12	F	63	NR	M1c	14.4	Deceased	29	Ipilimumab	Skin/chest wall metastasis
13	F	55	R	M1c	32.8	Deceased	24	Ipilimumab	Elbow metastasis
14	M	62	NR	M1c	64.8	Alive	47	Ipilimumab	Nonresponding adrenal gland metastasis

M, male; F, female; NR, nonresponse; R, response; ND, not documented; CNS, central nervous system.

### Why AR is a plausible therapy-responsive regulator in melanoma

Several lines of evidence support the plausibility of AR as a therapy-responsive regulator in melanoma. Prior studies suggest that AR signaling can be increased or become functionally relevant under pressure from targeted therapy in melanoma, including work showing that AR contributes to targeted-therapy resistance and that AR blockade can promote response to BRAFi/MEKi in preclinical models (27, 28). This positions AR as a plausible therapy-responsive transcriptional regulator, a key feature of any upstream driver model of adaptive resistance. We do not argue for universality; rather, we propose that AR may represent one axis of adaptive plasticity that becomes relevant in specific tumor states, treatment contexts, and microenvironmental conditions.

### Mechanistic precedent: B7-H3 regulation across tumors and a defined AR–B7-H3 link in prostate cancer

Key cross-tumor mechanisms reported to regulate B7-H3, together with the more defined AR–B7-H3 precedent in prostate cancer, are summarized schematically in Figure 1.

B7-H3 expression is not a static attribute; across tumor types, it is shaped by tumor-intrinsic signaling, adaptive stress responses, and epigenetic and post-transcriptional circuits. Mechanistic and integrative studies have implicated upstream control of B7-H3 by growth factor and stress pathways, including mTORC1-linked transcriptional control, as well as by epigenetic and microRNA-associated mechanisms that can uncouple RNA and protein output (29, 30).

In particular, mTORC1 can upregulate B7-H3 by p70S6K-dependent phosphorylation of the transcription factor YY2, providing a direct mechanistic link between mTORC1 hyperactivity and increased B7-H3 expression (29). In mTORC1-hyperactive settings, genetic or pharmacologic suppression of the mTORC1 → S6K axis reduces B7-H3 at both mRNA and protein levels and decreases B7-H3 promoter activity, supporting a transcriptional control layer downstream of mTORC1 (29). Mechanistically, YY2 binds the CD276 promoter, and S6K-dependent phosphorylation of YY2 at Thr336 stabilizes YY2 by limiting Smurf1-mediated ubiquitination and proteasomal degradation, thereby providing a concrete signal-to-transcription route for sustained B7-H3 upregulation under growth/metabolic stress (29).

A clear example of lineage-factor coupling comes from prostate cancer. Expression-based analyses support an association between B7-H3 levels and androgen-linked programs, including relationships with AR activation and immune/stromal features (31, 32). However, the directionality of this relationship appears to be context-dependent. AR ChIP data suggests the presence of an AR-binding site upstream of B7-H3, and androgen manipulation has been reported to modulate B7-H3 output in LNCaP cells, consistent with a potential direct regulatory component in some settings (31). At the same time, longitudinal patterns under androgen-deprivation therapy indicate that the AR–B7-H3 relationship can shift across disease stage (33).

Because AR can transcriptionally support amino acid influx required to sustain mTORC1 signaling, for example through regulation of LAT3-mediated leucine uptake (34), an AR → mTORC1 → YY2 pathway represents a plausible bridge between therapy-induced AR activity, nutrient sensing, and increased B7-H3 output. Consistent with this model, AR–mTOR crosstalk itself is influenced by testosterone availability. In LNCaP cells, AR positively regulates mTOR activity under both high- and low-testosterone conditions, whereas sub-basal mTOR activity can feed back to increase AR protein levels specifically under low testosterone conditions. Together, these findings support a bidirectional adaptive circuit that could tune mTORC1 output during androgen-limited stress (35).

By contrast, longitudinal androgen-deprivation models suggest that B7-H3 can increase early after androgen withdrawal and then become largely AR-irrelevant, whereas it tracks proliferative relapse later in castration-resistant progression (33), underscoring that the AR–B7-H3 relationship can invert across contexts and disease stages. Together, these precedents provide a framework for considering how therapy-induced AR activity in melanoma could engage shared upstream nodes (e.g., nutrient-sensing mTORC1 → YY2 control) to promote a B7-H3-high immune-excluded state, while keeping the directionality explicitly testable rather than assumed.

Beyond androgen signaling, defined transcriptional routes link common oncogenic lesions to B7-H3 upregulation. For example, concurrent PTEN/TP53 defects can activate Sp1 to directly drive B7-H3 transcription: Sp1 binds the B7-H3 promoter, and Sp1 perturbation reduces B7-H3 RNA and protein in PTEN/TP53-deficient models (36). As additional cross-tumor support, prior studies suggest that elevated mTOR signaling may be sufficient to induce B7-H3 expression in certain tumor contexts. MiT/TFE translocation RCC models show fusion-linked mTOR activation with coordinated B7-H3 upregulation, and pharmacologic (e.g., Torin1/RapaLink-1/RMC-5552) or genetic inhibition of mTOR signaling downregulates B7-H3 in inducible and tumor-derived systems (37).

Together, these regulatory routes nominate specific nodes that can be interrogated in melanoma models to test whether AR engages B7-H3 circuitry under treatment pressure.

### Mechanistic hypotheses: how AR activity could promote B7-H3–linked immune exclusion

We outline mechanistic hypotheses that are explicitly testable and aligned with established modes of B7-H3 regulation in other tumor settings.

First, AR may increase B7-H3 output by intersecting known regulatory circuitry at both transcriptional and translational levels (29, 30). Because AR and PI3K–AKT signaling can reciprocally compensate through feedback loops (38), treatment-adaptive AR activity could help sustain nutrient-sensing and translational programs that favor B7-H3 expression (36). In melanoma, BRAFi/MEKi or ICB-associated inflammatory stress could engage these same nodes, providing a testable path by which AR amplifies B7-H3 output in specific treatment-adapted states.

Second, AR-driven state transitions may dampen inflammatory sensing and antigen presentation, indirectly favoring B7-H3-high immune exclusion. If AR activity reduces interferon responsiveness, antigen presentation, or chemokine programs required for T-cell recruitment, tumors may become less visible to immune surveillance. Emerging evidence links B7-H3 biology to interferon/STAT1-associated circuits in the tumor microenvironment, providing a plausible bridge between tumor-intrinsic state and immune suppression (39).

Third, AR may contribute to barrier formation and stromal remodeling that phenocopies the collagen-rich architecture associated with B7-H3–high melanoma. Immune exclusion is frequently reinforced by extracellular matrix remodeling, fibroblast activation, and altered adhesion programs. AR-associated programs could promote or stabilize stromal and extracellular matrix features that restrict immune infiltration, consistent with the barrier-like phenotype observed in B7-H3–high melanoma (10).

Fourth, AR may synchronize immune exclusion with invasive and plasticity-associated traits. Therapy-resistant melanoma states often couple phenotypic switching with immune evasion (20–22). AR could participate in transcriptional programs that enhance migration and invasion while simultaneously promoting immune escape, yielding a coordinated resistant state in which B7-H3 serves as a tractable surface marker and potential functional mediator.

In this model, therapeutic pressure may increase AR activity in a subset of tumors, which could engage B7-H3 regulatory circuitry and state-transition programs, leading to a B7-H3-high immune-excluded architecture and reduced sensitivity to BRAFi/MEKi and/or ICB. Each link in this chain is falsifiable and requires causal testing.

### Translational opportunities: disrupting the axis

If AR contributes to maintaining a B7-H3–linked immune-excluded state, modulation of the AR pathway could be explored as a strategy to destabilize adaptive resistance, potentially in combination with BRAFi/MEKi or ICB. The rationale would not be that AR inhibition directly substitutes for B7-H3 targeting, but rather that upstream disruption of AR-dependent adaptive signaling might reduce the stability of the resistant state and thereby improve vulnerability to other therapies.

**Figure 2:**
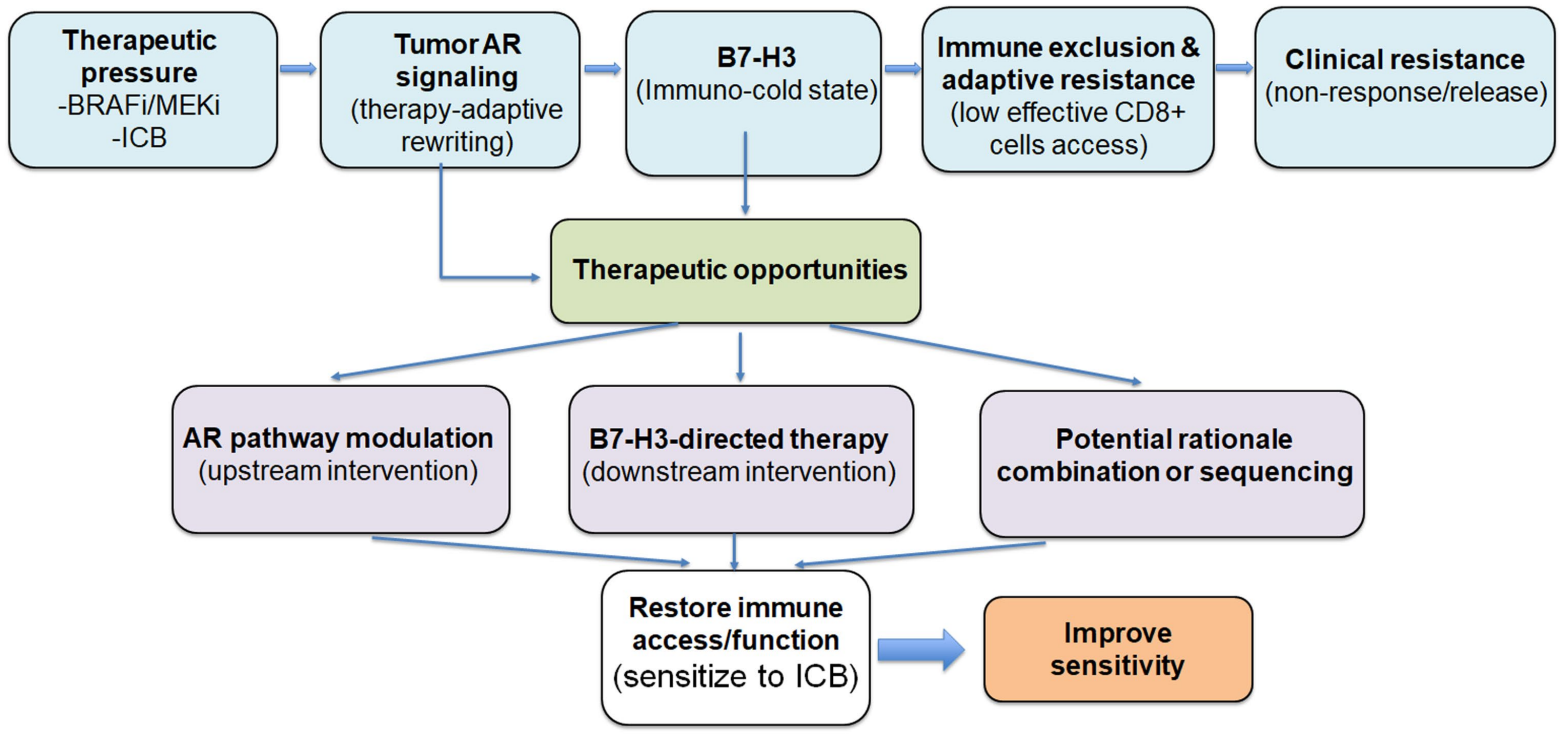
Proposed framework linking therapy-adaptive AR signaling to a B7-H3–associated immune-exclusion program in melanoma. Therapeutic pressure from BRAF/MEK inhibitors or immune checkpoint blockade may increase tumor-intrinsic AR signaling in a subset of melanomas. In this hypothesis-generating model, adaptive AR activity contributes to induction or stabilization of a B7-H3-high immune-cold state associated with immune exclusion, adaptive resistance, and reduced treatment sensitivity. The lower portion of the schematic highlights potential therapeutic intervention points, including AR pathway modulation as an upstream strategy and B7-H3-directed therapy as a downstream strategy. These therapeutic approaches are presented as distinct but potentially complementary, rather than as a fixed linear treatment sequence.

In parallel, B7-H3-directed therapeutic approaches continue to expand across oncology, and multiple modalities (including antibodies, ADCs, bispecifics, and cell-based therapies) offer practical approaches to target B7-H3-high tumor states. A central translational question is whether B7-H3-high melanoma represents a therapeutically distinct subgroup that requires direct B7-H3 targeting, upstream disruption through AR modulation, or both. By explicitly separating an upstream candidate driver (AR) from a downstream immune-evasion node (B7-H3), this framework motivates rational combinations and suggests practical engagement readouts, including reduced B7-H3 output and reversal of immune-exclusion-associated measures (Figure 2).

### Testable predictions and near-term study designs

To move from hypothesis to validation, this framework yields specific, testable predictions. It predicts that (i) AR activity will increase in a subset of post-treatment or resistant tumors; (ii) B7-H3 will rise in parallel with AR during therapy adaptation in the same subset; (iii) AR-high/B7-H3-high tumors will show immune exclusion signatures (e.g., reduced intratumoral CD8 density, dampened interferon/antigen-presentation programs, and stromal/ECM remodeling features); and (iv) genetic or pharmacologic AR perturbation will reduce B7-H3 output and exclusion-associated phenotypes in model systems. These predictions can be evaluated using paired pre- and on-treatment tissue analyses, treatment-conditioned melanoma models (2D and 3D), and immune co-cultures designed to measure both tumor-intrinsic B7-H3 regulation and functional immune engagement.

This framework also raises the possibility that immunocompetent animal models could help define whether AR- and B7-H3-linked immune exclusion contributes to sex-biased therapeutic responses in melanoma. Prior longitudinal studies in spontaneous melanoma mouse models have demonstrated therapy-associated sex differences in treatment response, survival, and tumor–microenvironment composition, suggesting that such systems may provide an informative platform for testing whether therapy-adaptive AR signaling, B7-H3 expression, and immune exclusion diverge by sex and together shape response to targeted therapy or immune checkpoint blockade.

More broadly, a systematic evaluation of the relationship among AR signaling, B7-H3 expression, immune exclusion, and treatment response may help identify promising therapeutic strategies for early-phase clinical testing in patients with treatment-refractory melanoma. This may be particularly relevant early in the course of therapy, when multiple treatment options remain available, and pre-treatment or early on-treatment biopsies could help identify tumor phenotypes that might be manipulated to prevent or overcome resistance in both immune checkpoint blockade and targeted therapy settings.

Study designs to address these predictions are summarized in Table 2.

**Table 2:** Translational roadmap for evaluating an AR–B7-H3 immune-exclusion framework in melanoma.

Key hypothesis/question	Core readouts (examples)	Specimens/models	Suggested approach	Translational utility
Therapy induces tumor AR signaling	AR IHC/IF (nuclear); AR signature score	Pre−/on-treatment biopsies; melanoma lines under BRAFi/MEKi	Paired biopsy comparisons; treatment-conditioned models	Identifies a therapy-responsive upstream candidate driver
AR-high associates with increased B7-H3	B7-H3 RNA/protein; B7-H3 flow/IHC	Human tumors; melanoma lines	Stratify by treatment status; correlate AR vs. B7-H3 across states	Defines AR–B7-H3 coupled resistant states
AR causally regulates B7-H3 output	B7-H3 after AR knockdown/overexpression; AR antagonists	Melanoma lines; 3D spheroids/organoids	Orthogonal perturbations (genetic + pharmacologic) with time-course profiling	Establishes directionality for targeting
AR–B7-H3 states exhibit immune exclusion	CD8 density; interferon response; antigen presentation; ECM/stromal features	Tumor tissue; spatial profiling	Immune phenotyping and spatial analysis in AR-high/B7-H3-high tumors	Links tumor state to immune-cold architecture
AR modulation sensitizes to therapy	Restored inflammatory programs; improved immune killing in co-culture	Tumor–immune co-cultures; 3D models	AR modulation ± PD-1 blockade or BRAFi/MEKi	Supports a combination rationale
B7-H3 targeting complements ICB in resistant-state models	Tumor control; immune activation readouts	Preclinical systems (3D; *in vivo* where feasible)	Anti–B7-H3 ± anti–PD-1 in B7-H3-high state models	Enables state-directed immunotherapy

Summary of key testable hypotheses, representative readouts, suggested specimen sources and model systems, and near-term study designs to validate AR–B7-H3 coupling, define immune-excluded resistant states, and enable state-directed therapeutic strategies. IHC, immunohistochemistry; IF, immunofluorescence; ECM, extracellular matrix; ICB, immune checkpoint blockade.

## Discussion and outlook

We propose AR signaling as a candidate upstream driver linking therapeutic pressure to a B7-H3–associated immune-excluded resistance state in melanoma. The central contribution of this framework is not the assertion that AR universally controls B7-H3 in melanoma, but rather the articulation of a falsifiable chain of events that connects therapy-induced AR activity to established B7-H3 regulatory circuitry and to a clinically recognizable immune-excluded architecture. This hypothesis is grounded in both a therapy-associated clinical correlation signal and mechanistic precedent from other tumor contexts in which B7-H3 is regulated by defined signaling and epigenetic programs (29–33, 36, 39).

The near-term value of this framework lies in its practicality: it defines measurable predictions, identifies therapeutic nodes (AR and B7-H3), and provides a concise validation roadmap (Figure 2, Table 2). Soluble B7-H3 has been explored in other tumors (40–43), but its relevance to melanoma state transitions remains unknown. If validated, the framework could support state-guided combination strategies to prevent or reverse immune exclusion during treatment and improve response durability in a subset of patients.

## Data Availability

The original contributions presented in the study are included in the article/supplementary material, further inquiries can be directed to the corresponding author.
